# Reply to: Biological role of miR-204 and miR-211 in melanoma (published in Oncoscience, Vol 5 (7-8), July 2018)

**DOI:** 10.18632/oncoscience.473

**Published:** 2018-11-09

**Authors:** Marta Díaz-Martínez, Lucía Benito-Jardón, Lola Alonso, Joaquin Teixidó

**Affiliations:** Department of Cellular and Molecular Medicine, Centro de Investigaciones Biológicas (CSIC), 28040 Madrid, Spain.

**Keywords:** mmelanoma, resistance, microRNA

Melanoma treatment with the BRAF V600E inhibitor vemurafenib provides therapeutic benefits, but the frequent emergence of drug resistance remains an important clinical challenge. Although reactivation of the MAPK and PI3K–Akt pathways is a common resistance response in melanoma patients treated with vemurafenib, a significant portion of tumors displays unknown resistance mechanisms that cannot be accounted for genetic alterations. In a recent study we addressed the potential role of microRNAs in resistance to vemurafenib in melanoma, and we showed that rapid upregulation of miR-204-5p and miR-211-5p following vemurafenib treatment enables the emergence of early resistance. These conclusions were experimentally supported by ectopic expression of these miRNAs in drug-naïve human melanoma cells, which was sufficient to confer vemurafenib resistance and more robust tumor growth *in vitro* and *in vivo*. Conversely, silencing the expression of these miRNAs in vemurafenib-resistant cells impaired cell growth in the presence of the inhibitor. In a recent short report by *Vitiello and colleagues* published in Oncoscience, authors claimed that conclusions of our work were not supported by the published data. In the present answer, we provide a reasoned explanation that confirms and supports our results.

We recently reported that miR-204-5p and miR-211-5p are upregulated in melanoma cells resistant to vemurafenib (VMF), and that these miRNAs are involved in early phases of melanoma resistance to (VMF) [1]. The analysis of miRNA alterations was initially performed using small RNA-seq from A375 parental and VMF-resistant (A375-VR) cells, and changes in miRNA expression were subsequently confirmed by real-time quantitative PCR. To analyze miRNA expression we used the miRNA-specific TaqMan MicroRNA Assay Kits (Applied Biosystems), a widely used tool to analyze the expression of specific miRNAs. After reverse-transcribing RNA with the TaqMan microRNA Reverse Transcription Kit (Applied Biosystems), quantitative PCR was performed in triplicate with corresponding TaqMan PCR primers (Applied Biosystems), and TaqMan Universal Master Mix. According to the manufacturer, this system allows efficient detection of specific miRNAs, even with similar sequences. With this method we confirmed upregulated expression of miR-204-5p and miR-211-5p, amongst several additional alterations, in A375-VR cells as well as in parental A375 cells treated with VMF. Parental A375 cells showed low levels of miR-211-5p in the absence of MAPK inhibitors, as also observed by *Vitiello et al* [2], and its expression was increased after drug treatment, which was maintained in the VMF-resistant cells [1]. Moreover, expression of miR-204-5p and miR-211-5p was also enhanced in the BRAF V600E melanoma cell line SK-Mel-28 after treatment with VMF, as well as in 5 out of 6 VMF-resistant clones of the BRAF V600E melanoma cell line SK-Mel-239. In addition, altered expression of these miRNAs was also observed in cells treated with trametinib (TMT), or with combined VMF and TMT, whereas no changes were detected with Akt and Rac inhibitors. Our data are therefore in line with previous reports on the upregulated miR-204-5p and miR-211-5p expression in melanoma cells exposed to vemurafenib [2-4].

To confirm the specificity of these changes, we demonstrated that normal foreskin melanocytes exposed to VMF displayed increased miR-204-5p expression, whereas no significant changes in miR-211-5p expression were detected [1] (Supplementary Fig. S4D). We also used SK-Mel-103 cells to test the effect of VMF in an inherently VMF-resistant melanoma cell line. The results revealed that miR-204-5p expression was augmented by VMF, whereas no miR-211-5p expression was detected in these cells. Our results confirm the specificity of the probes tested for specific detection of miR-204-5p or miR-211-5p, and do not support the claims and concerns raised by *Vitiello et al* [5].

To analyze the role of miR-204-5p and miR-211-5p in melanoma resistance to VMF, we used two different approaches. We separately overexpressed these miRNAs in A375 and SK-Mel 28 parental cells, or silenced them in A375-VR cells. Using two different proliferation assays, we found that miRNA overexpression leads to a moderate but consistent increase in resistance to VMF, as well as to combined VMF and TMT, but not to the ERK inhibitor SCH772984. These results indicate that miR-204-5p and miR-211-5p overexpression provides survival advantage against vemurafenib. Moreover, to add significance to our data, we showed that silencing miR-204-5p and miR-211-5p in A375-VR cells causes a decrease in the growth of stably-transduced cells in the presence of VMF, highlighting the involvement of these miRNAs in resistance to this inhibitor. In their short report, *Vitiello et al* [5] seem to inadvertently missed our overexpression data.

As miR-211-5p is expressed from the intron 6 of *TRPM1*, we decided to analyze if the expression levels of *TRPM1* itself were also increased in A375-VR, using for this purpose the described primers (Supplementary Table S1B) [1]. The design of the oligonucleotides was performed using standard procedures with the NCBI tool Primer-BLAST (considering the size of the product amplified, melting temperatures of the two primers, GC content and unintended products), with the final aim of examining *TRPM1* expression, whereas for the detection of miR-211-5p we specifically used TaqMan MicroRNA Assay Kits (Applied Biosystems). Data revealed that the increased miR-211-5p expression in A375-VR cells directly correlated with enhanced expression TRPM1 relative to parental cells.

Collectively, our results clearly show that miR-204-5p and miR-211-5p are overexpressed in VMF-resistant melanoma cells and contribute to early phases of melanoma resistance to VMF. Examination of some potential targets for these miRNAs considered to be of interest for our investigations revealed that the NUAK1/ ARK5 protein was consistently reduced in VMF-resistant cells and in transductants overexpressing miR-211-5p, but not in those overexpressing miR-204-5p, again highlighting important differences between these two miRNAs. Based on published data [6, 7], our results raise the possibility that NUAK1 could be involved in VMF resistance in melanoma cells. Therefore, our conclusions are supported by strong and carefully analyzed data, and shed doubts about the claims and concerns raised by *Vitiello and colleagues* in their short report [5].
